# Skin thickness alterations in pressure injury tissue: insights from high-frequency ultrasound and mixed-design analysis of variance

**DOI:** 10.3389/fphys.2026.1824326

**Published:** 2026-06-03

**Authors:** Lin Tang, Jingmin Zhang, Wu Zhou, Jing Huang, Dongmei Diao, Luying Zhong, Jianna Zhang

**Affiliations:** 1 Department of Emergency Medicine, West China Hospital, Sichuan University/West China School of Nursing, Sichuan University, Chengdu, Sichuan, China; 2 Department of Ultrasound Medicine, West China Hospital, Sichuan University/West China School of Nursing, Sichuan University, Chengdu, Sichuan, China

**Keywords:** nursing assessment, patient safety, pressure injury, risk stratification, skin thickness measurement, ultrasonography

## Abstract

**Purpose/aim:**

To quantify skin thickness alterations in pressure injury (PI) tissue and evaluate the influence of PI risk factors using high-frequency ultrasound.

**Background:**

Early PI detection remains challenging due to limitations of visual assessment. High-frequency ultrasound (HFUS) offers high-resolution skin imaging but lacks validation in PI tissues.

**Design:**

Prospective comparative study.

**Methods:**

In this cross-sectional study, we assessed skin thickness in 42 bedridden patients with pressure injuries (PIs). For each patient, measurements were taken at the PI site and an adjacent normal site using high-frequency ultrasound (18 MHz). A mixed-design ANOVA tested the effects of tissue type (within-subject: PI vs. normal), PI risk status (between-subject: high vs. low), and body mass index (BMI: ≥24 vs.<24 kg/m²), including all main and two-way interaction effects. *Post-hoc* tests were Bonferroni-corrected, and effect sizes were quantified using generalized eta-squared (η²G).

**Results:**

Skin at pressure injury (PI) sites was substantially thicker than at adjacent normal sites (3.26 ± 0.63 mm vs. 2.11 ± 0.52 mm). A mixed-design ANOVA confirmed a large, significant main effect for tissue type (F (1, 38) = 57.980, p< 0.001, $\eta_{g}^{2}=0.500$). Additionally, the model revealed a significant main effect for PI risk status, with high-risk patients exhibiting greater overall skin thickness (F (1, 38) = 11.150, p = 0.002, $\eta_{g}^{2}=0.090$). The main effect of body mass index was not significant (p = .510). While no interactions reached statistical significance, the interaction between tissue type and PI risk status trended towards significance (F (1, 38) = 3.20, p = .082, $\eta_{g}^{2}=0.050$). This trend reflected a pattern wherein the difference in skin thickness between PI and normal sites was more pronounced in high-risk patients compared to low-risk patients. No other interactions were significant (all p > 0.050). All model assumptions were met.

**Conclusions:**

HFUS-detected skin thickening is a hallmark of PI tissue, primarily driven by local pathology and modulated by systemic risk factors. HFUS shows potential for objective PI assessment.

## Introduction

Pressure injuries (PIs), localized damages to the skin and underlying tissues from sustained pressure or shear, represent a significant clinical burden associated with high morbidity and mortality (Baron et al., 2022; Nghiem et al., 2022; Yang et al., 2025). The severe consequences of PIs underscore a critical need for accurate, early assessment to guide effective staging and treatment (Hillier et al., 2025; Tusar et al., 2025).

Current clinical assessment relies heavily on visual skin assessment (VSA) based on the National Pressure Injury Advisory Panel (NPIAP) staging system (National Pressure Injury Advisory Panel et al., 2025; Wei et al., 2025). Nevertheless, VSA is subjective and often fails to detect deep tissue damage before it becomes visible, a limitation exacerbated in patients with diverse skin tones (Cu et al., 2025; Ho et al., 2025). Although MRI offers superior soft-tissue contrast, its routine use is constrained by cost, complexity, and immobility (Hori et al., 2021; Thompson et al., 2021). Other technologies, such as finite element analysis (FEA) and subepidermal moisture (SEM) measurement, remain either too theoretical for bedside use or do not fully capture deep tissue changes (Hiscox et al., 2025; Lin et al., 2025). This technological gap contributes to significant diagnostic inaccuracies, highlighting an urgent need for more reliable methods.

High-frequency ultrasound (HFUS) is emerging as a promising solution. As a non-invasive, portable, and radiation-free tool, HFUS provides real-time, high-resolution images of skin layers with a precision approaching histopathology (Dias et al., 2024; Wu et al., 2025). Its utility is enhanced when combined with shear wave elastography (SWE) for quantitative assessment of tissue mechanics, and its value in PI management is increasingly supported by evidence (Kijanka and Urban, 2021; Yin et al., 2024). Recent studies (Liu et al., 2024; Hillier et al., 2025; Naylor and Velthuis, 2025) have demonstrated HFUS’s ability to quantitatively assess tissue changes in high-risk patients and outperform traditional risk scales in predicting hospital-acquired PIs. Furthermore, standardized ultrasound patterns are being developed to objectively identify deep tissue injury (Landolfo et al., 2025; Palermi et al., 2025).

To address these gaps, the present study employs HFUS to conduct a systematic investigation into skin thickness alterations in PI tissue (Wang et al., 2025) We seek not only to quantify the differences between PI sites and adjacent normal tissue but also to understand how these structural changes are modulated by the interplay of tissue type and patient-specific factors. It is anticipated that these findings will provide a foundation for establishing robust imaging biomarkers for early PI detection and guiding the development of more personalized care strategies.

## Materials and methods

### Study design and participants

This prospective study was conducted at West China Hospital, Sichuan University, from January 2025 to April 2025, following approval by the hospital’s Institutional Review Board. After obtaining written informed consent, we enrolled 42 bedridden patients with PIs, analyzing 84 tissue sites in a within-subject comparative design. Each patient’s PI site was compared to their own adjacent healthy tissue, serving as a self-control. The sample size was calculated to achieve over 80% statistical power for detecting medium-to-large effect sizes with our planned mixed-design ANOVA.

### Participant select

Individuals with scars, surgical history, or soft tissue injury at the relevant sites did not meet the inclusion criteria from the outset and were therefore excluded. Inclusion criteria for study participants: ① Patients aged 18 or older in the emergency department resuscitation room whose condition is stable; ② PI of the sacrococcygeal region diagnosed as Stage I/II by two wound care nurses with more than five years of wound management experience by NPIAP 2025 guideline; ③ Intact PI and surrounding skin, with a distinct area of healthy, uninvolved skin 3–5 cm from the PI border for intra−patient comparison; ④Informed consent provided by the patient or their legal representative. Exclusion criteria: ①Confounding skin conditions such as eczema or psoriasis at the measurement site; ② Presence of active infection, severe necrosis, or heavy exudate in the PI; ③ Cognitive impairment or medical conditions preventing adequate cooperation with data collection.

### High-frequency ultrasound examination

#### Imaging protocol

**Figure 1:**
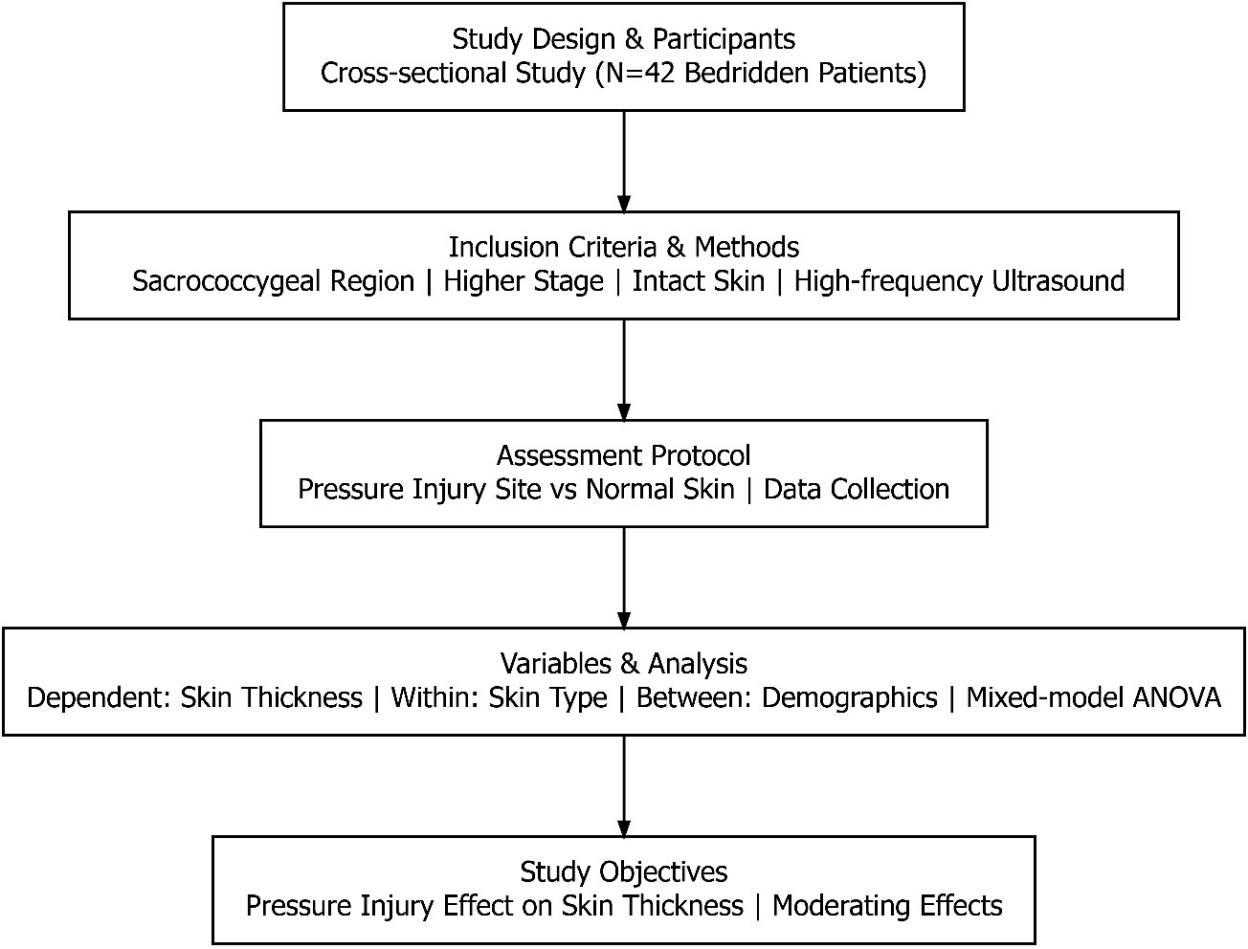
Study design flowchart. The study enrolled 42 bedridden patients with buttock/sacral pressure injuries of higher stages and intact skin. High-frequency ultrasound was used to compare skin thickness between pressure injury sites and normal skin. A mixed-model ANOVA was applied to analyze the effects of skin type (within-subject factor) and demographic variables (between-subject factors) on skin thickness, aiming to evaluate pressure injury-related changes and potential moderating effects.

Skin thickness was assessed using a portable color Doppler ultrasound system (Mindray M9, Mindray Bio-Medical Electronics, China) with an 18 MHz high-frequency linear array probe (axial resolution: 0.1 mm) or a Philips CX50 system (Philips Healthcare, Netherlands) with an L12–3 linear probe, depending on availability. To ensure consistency, both systems were standardized to the musculoskeletal imaging preset with a fixed depth of 3.0 cm and optimized gain settings (**Figure 1**). The skin thickness in this study refers to the sum of the thickness of the epidermal layer and the thickness of the dermis layer. Detailed identification and description of skin layers, including epidermis, dermis, superficial adipose tissue, superficial fascia, and deep fascia, as detailed in the team’s previously published research (Wang et al., 2025).

#### Measurement procedure

All examinations are performed by a certified, specialized ultrasound technologist with over 10 years of experience (including at least 5 years in musculoskeletal imaging) who has received specialized training for this examination protocol. Participants were placed in a stable lateral or prone position to ensure optimal access to the measurement sites.

Two types of sites were marked: the center of the PI lesion and four points on adjacent tissue (cephalad, caudad, left, and right) located 3–5 cm from the PI margin. The adjacent tissue was confirmed as normal by visual inspection, palpation, and sonographic appearance. For each measurement, the probe was held perpendicular to the skin with minimal pressure to prevent tissue compression. An image was acquired when the hyperechoic epidermis, the uniformly medium-echoic dermis, and the dermal-subcutaneous junction were clearly demarcated.

Skin thickness was measured as the vertical distance from the outer epidermal surface to the dermal-subcutaneous junction using built-in electronic calipers. To ensure reliability, three separate measurements were taken at each marked site with slight probe repositioning, and the mean value was used for analysis.

#### Data collection and definitions

Demographic and clinical data were collected prospectively, including age (categorized as ≤50 vs. >50 years), sex, and Body Mass Index (BMI); categorized as ≤24 vs. >24 kg/m²). A composite PI risk status was defined according to previous literature (Alderden et al., 2025). Consistent with established PI risk prediction scales, participants were classified as high-risk if they presented with one or more of the following pre−existing comorbidities known to elevate PI risk and impede tissue repair: diabetes mellitus, chronic kidney disease, heart failure, rheumatoid arthritis, or systemic lupus erythematosus. Participants were classified as low−risk if none of these comorbidities were present. Accurate risk identification requires integration of comorbidity−based risk stratification and comprehensive clinical judgment, rather than reliance on risk assessment scales alone (Hillier et al., 2025).

### Statistical analysis

Skin thickness was analyzed using mixed-design ANOVA in R 4.5.2. Candidate variables initially considered for inclusion included tissue type, age, gender, BMI, and PI risk status. Variable selection was guided by clinical relevance, variance inflation factor (VIF< 5) to exclude multicollinearity, and model fit statistics to derive a parsimonious model and avoid singularity. The final model included tissue type as a within-subject factor, and PI risk status and BMI as between-subject factors.

The mixed-design ANOVA tested all main effects and two-way interactions using Type III sums of squares. Effect sizes were quantified with generalized eta-squared ( $\eta_{g}^{2}$). Significant interactions were probed with Bonferroni-corrected simple effects analysis on the estimated marginal means. *Post-hoc* power for the observed interaction was also calculated. Model validity was confirmed through standard diagnostic checks of residuals. All tests were two-tailed, with statistical significance set at α = 0.05.

## Results

### Participant characteristics and baseline associations

A total of 42 bedridden patients were enrolled, from whom 84 paired skin tissue observations (PI and adjacent normal tissue) were collected, all skin tissue samples were collected at the sacrococcygeal region, and all PI are classified as stage II. The study population was predominantly male (74%) and older (81% > 50 years, **Table 1**), with an equal distribution between high and low PI risk status (n = 21 each). The criteria for high-risk status were heterogeneous, with chronic kidney disease being the most frequent comorbidity (50%), often in combination with heart failure or diabetes. Detailed cohort demographics are presented in **Table 2**.

**Table 1:** Descriptive statistics of study variables.

Characteristic	N = 42^1^
Gender	
Female	11 (26%)
Male	31 (74%)
Age	
< 50	8 (19%)
≥ 50	34 (81%)
Body Mass Index	
< 24	30 (71%)
≥ 24	12 (29%)
Pressure Injury Risk Status	
High risk^2^	21 (50%)
Low risk	21 (50%)
Skin Thickness	2.05 (1.60, 2.30)

^1^ n (%); Median (Q1, Q3);.

^2^ presence of one or more comorbidities.

**Table 2:** Distribution of pressure injury risk status among study participants.

Pressure injury risk status	N	Prop
	21	50.000
Chronic Kidney Disease	2	4.800
Chronic Kidney Disease + Heart Failure	3	7.100
Diabetes Mellitus	3	7.100
Diabetes Mellitus + Chronic Kidney Disease	1	2.400
Diabetes Mellitus + Chronic Kidney Disease + Heart Failure	4	9.500
Diabetes Mellitus + Heart Failure	1	2.400
Heart Failure	4	9.500
Rheumatoid Arthritis	1	2.400
Rheumatoid Arthritis + Heart Failure	1	2.400
Systemic Lupus Erythematosus	1	2.400

Preliminary correlational analysis revealed a strong positive association between tissue type andskin thickness (*r* = 0.847, *p* < 0.001) and a significant negative correlation between thickness and risk status (*r* = -0.286, *p* < 0.050). The complete correlation matrix is presented in **Supplementary Figure 1**.

### Increased skin thickness as a hallmark of pressure injury

**Figure 2:**
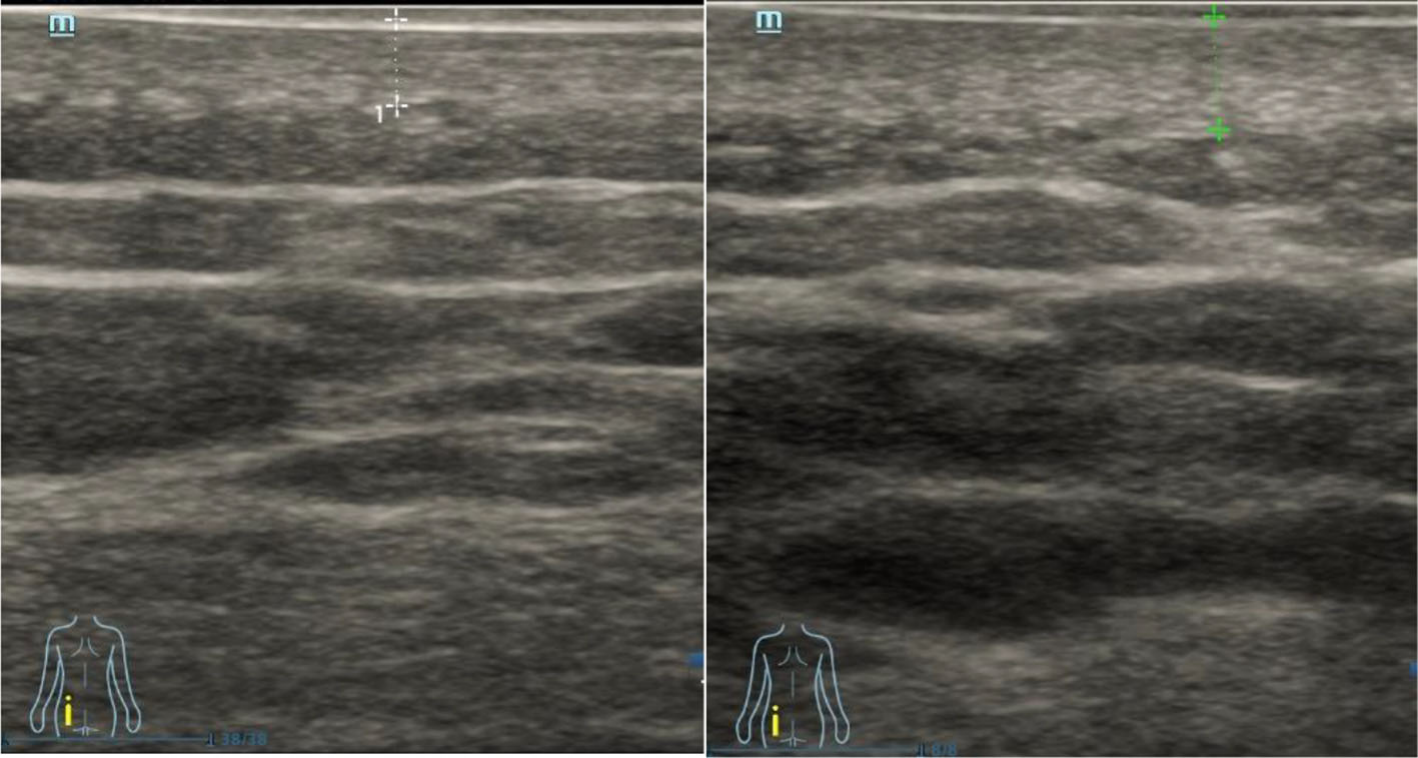
Ultrasound images of normal skin tissue (left, 2.9 mm) and pressure injury tissue (right, 3.7 mm) in the sacrococcygeal region of the same patient.

**Figure 3:**
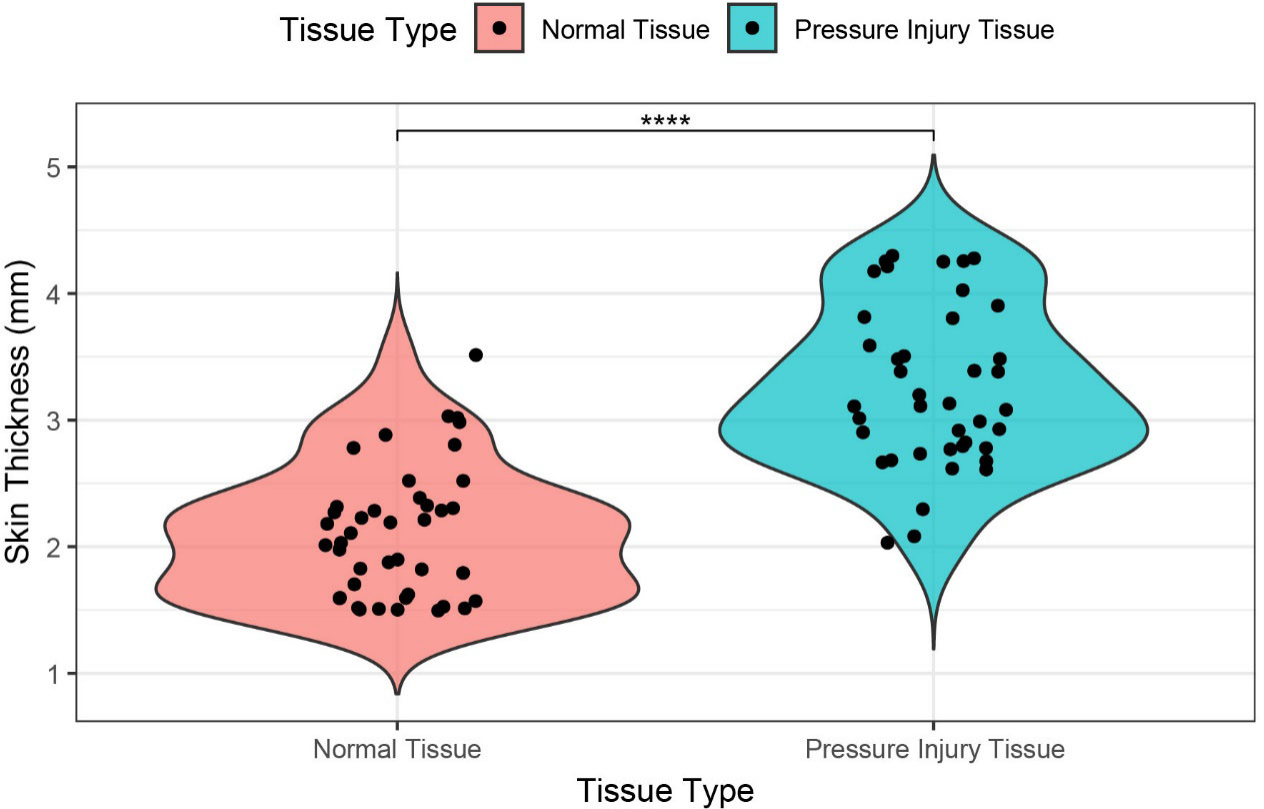
The violin plot illustrates the distribution of skin thickness (mm) for normal tissue and pressure ulcer tissue. Each violin shape represents the density of skin thickness measurements, with wider sections indicating a higher concentration of data points. A paired t-test was conducted to compare skin thickness between the two tissue types, with significant differences indicated on the plot. The violin plot illustrates the distribution of skin thickness (mm) for normal tissue and pressure ulcer tissue. Each violin shape represents the density of skin thickness measurements, with wider sections indicating a higher concentration of data points. A paired t-test was conducted to compare skin thickness between the two tissue types, with significant differences indicated on the plot. **** Average thickness difference.

The primary analysis revealed a pronounced and statistically significant increase in skin thickness at PI sites compared to adjacent normal tissue. On average, PI tissue was 1.15 mm thicker than matched normal tissue (3.26 [*SD* = 0.63] mm vs. 2.11 [*SD* = 0.52] mm; p< 0.001, **Figure 2**, **Table 3**). This difference corresponded to a very large effect size (*Cohen’s d* = − 1.990), underscoring the magnitude of this anatomical alteration. These aggregate findings are visually corroborated by **Figure 3**, where the violin plot illustrates not only a clear separation in the central tendency but also greater measurement variability within the PI tissue.

**Table 3:** Paired t-Test results comparing skin thickness between tissue types.

Characteristic	Normal tissue N = 42^1^	Pressure ulcer tissue N = 42^1^	Cohen's d	95%CI	p-value^2^
Skin Thickness	2.11 (0.52)	3.26 (0.63)	-1.99	-2.63, -1.54	<0.001

^1^ Mean (SD).

^2^ Paired t-test.

**Figure 4:**
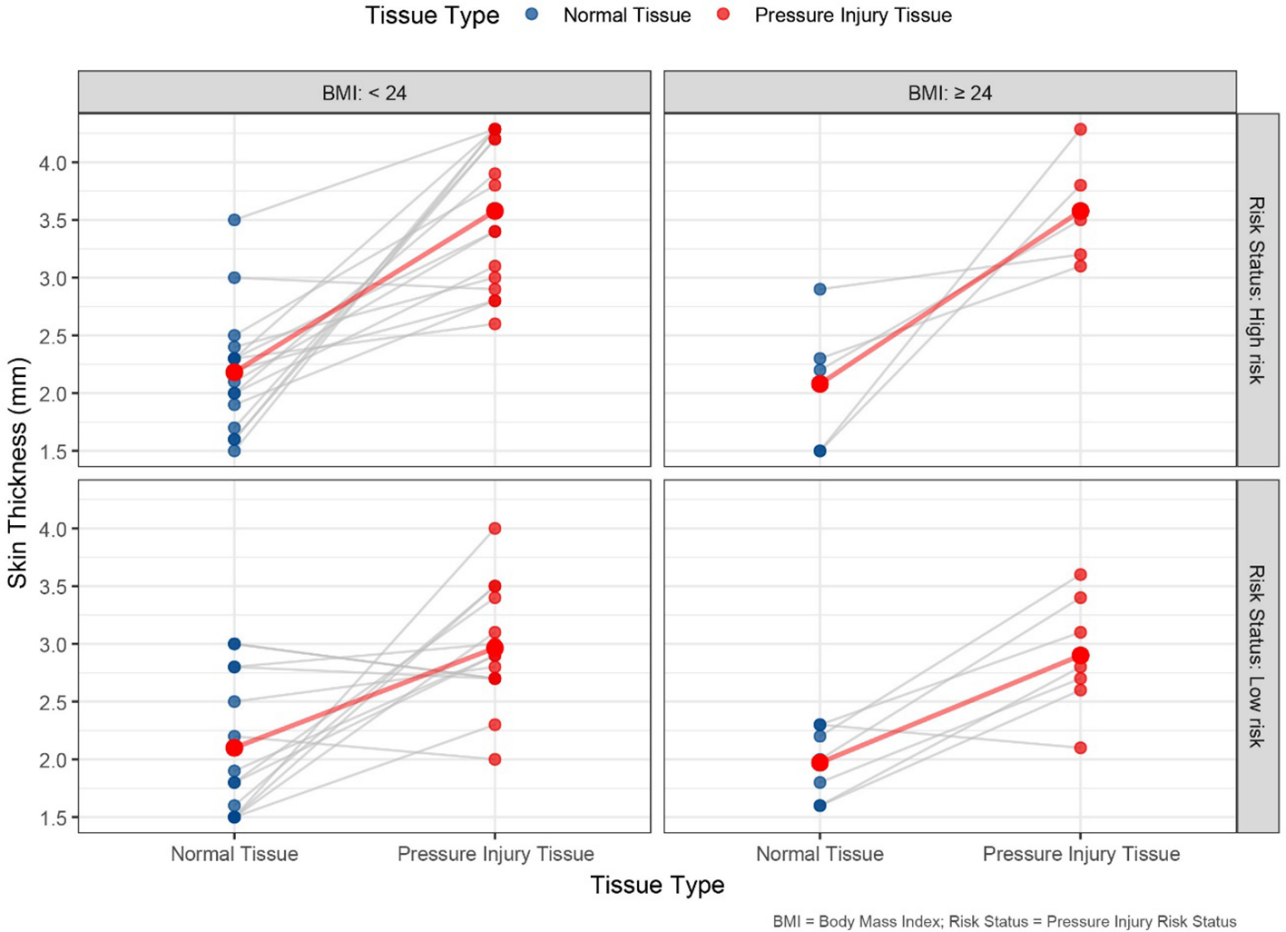
The spaghetti plot illustrates individual trajectories of skin thickness (mm) across normal tissue and pressure ulcer tissue. Each gray line represents an individual patient’s skin thickness change between the two tissue types, while colored points indicate measurements for each tissue type. Red points and lines denote the mean skin thickness across all individuals for each tissue type. The spaghetti plot illustrates individual trajectories of skin thickness (mm) across normal tissue and pressure ulcer tissue. Each gray line represents an individual patient’s skin thickness change between the two tissue types, while colored points indicate measurements for each tissue type. Red points and lines denote the mean skin thickness across all individuals for each tissue type.

To assess the consistency of this finding, we examined individual patient trajectories (**Figure 4**). Remarkably, every participant in the cohort (n=42) exhibited an increase in skin thickness at the PI site, without exception. This universal pattern of tissue thickening held true even when data were stratified by BMI or PI risk status, demonstrating that this phenomenon is a robust and fundamental characteristic of pressure injury, independent of these key patient factors.

### Modeling the determinants of skin thickness

**Figure 5:**
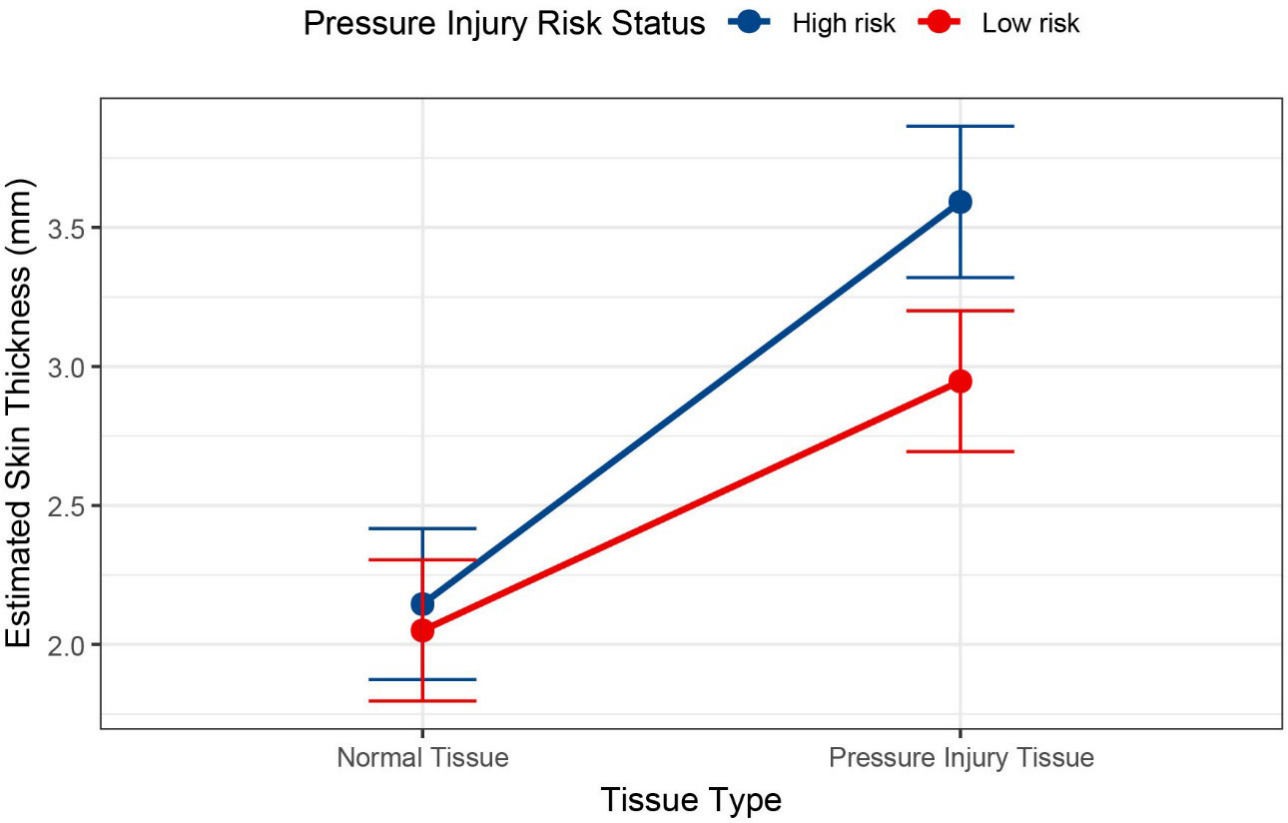
Interaction plot of tissue type and risk status on skin thickness. The interaction plot illustrates the estimated skin thickness (mm) across tissue types (normal vs. pressure ulcer) stratified by pressure injury risk status (high vs. low). Error bars represent 95% confidence intervals.

To identify factors contributing to skin thickness, a mixed-design ANOVA was performed. The results indicated significant main effects for Tissue Type (*F*(1, 38) = 57.98, *p* < 0.001, $\eta_{g}^{2}=0.500$) and PI Risk Status (*F*(1, 38) = 11.150, *p* = 0.002, $\eta_{g}^{2}=0.090$, **Table 4**), whereas the main effect of BMI was not significant (*p* = 0.510). An interaction between Tissue Type and PI Risk Status approached statistical significance (*F*(1, 38) = 3.200, *p* = 0.082, $\eta_{g}^{2}=0.050$). To further investigate this trend, a two-way repeated-measures ANOVA confirmed a similar interaction pattern (*F*(1, 40) = 3.970, *p* = 0.053, $\eta_{g}^{2}=0.061$, **Table 5**). Subsequent simple effects analysis revealed that skin was significantly thicker over PIs compared to intact skin within both the low-risk (mean difference = 0.90 mm, *p* < 0.001) and high-risk groups (mean difference = 1.45 mm, *p* < 0.001) (**Figure 5**, **Table 6**).

**Table 4:** Mixed-design ANOVA results.

Effect	DFn	DFd	F	p^1^	η²_g_^2^
(Intercept)	1.000	38.000	2,320.032	0.000***	0.954
Body Mass Index	1.000	38.000	0.442	0.510	0.004
Pressure Injury Risk Status	1.000	38.000	11.148	0.002**	0.091
Tissue Type	1.000	38.000	57.978	0.000***	0.501
Body Mass Index: Pressure Injury Risk Status	1.000	38.000	0.042	0.838	0.000
Body Mass Index: Tissue Type	1.000	38.000	0.072	0.790	0.001
Pressure Injury Risk Status: Tissue Type	1.000	38.000	3.197	0.082	0.052
Body Mass Index: Pressure Injury Risk Status: Tissue Type	1.000	38.000	0.004	0.953	0.000

^1^ *p< 0.05, **p< 0.01, ***p< 0.001;

^2^ η²_g_: Generalized Eta-squared based on Type III ANOVA.

**Table 5:** Results of repeated measures ANOVAs examining the effects of tissue type and individual between-subject factors.

Characteristic	Overall N = 84^1^	Normal tissue N = 42^1^	Pressure ulcer tissue N = 42^1^	P-Value^2^	η²_G_
Pressure Injury Risk Status				0.053	0.061
High risk	2.85 (2.20, 3.50)	2.20 (1.70, 2.30)	3.50 (3.10, 4.20)		
Low risk	2.65 (1.90, 3.00)	1.90 (1.60, 2.30)	2.90 (2.70, 3.40)		
Body Mass Index				0.956	0.000
< 24	2.75 (2.00, 3.25)	2.05 (1.60, 2.50)	3.10 (2.80, 3.90)		
≥ 24	2.45 (2.05, 3.15)	2.10 (1.60, 2.30)	3.15 (2.75, 3.55)		

^1^ Skin Thickness: Median (Q1, Q3).

^2^ Based on Mixed-design ANOVA; P and generalized η² values for interaction effects between Tissue Type and Between-Subject Factors.

**Table 6:** Simple effects analysis results.

Contrast	Pressure injury risk status	Estimate	Se	df	t	P-Value
Normal Tissue - Pressure Ulcer Tissue	High Risk	-1.447	0.228	38.000	-6.336	<0.001***
Normal Tissue - Pressure Ulcer Tissue	Low Risk	-0.896	0.206	38.000	-4.345	<0.001***

*p< 0.05, **p< 0.01, ***p< 0.001.

### Model diagnostics and assumption checks

**Figure 6:**
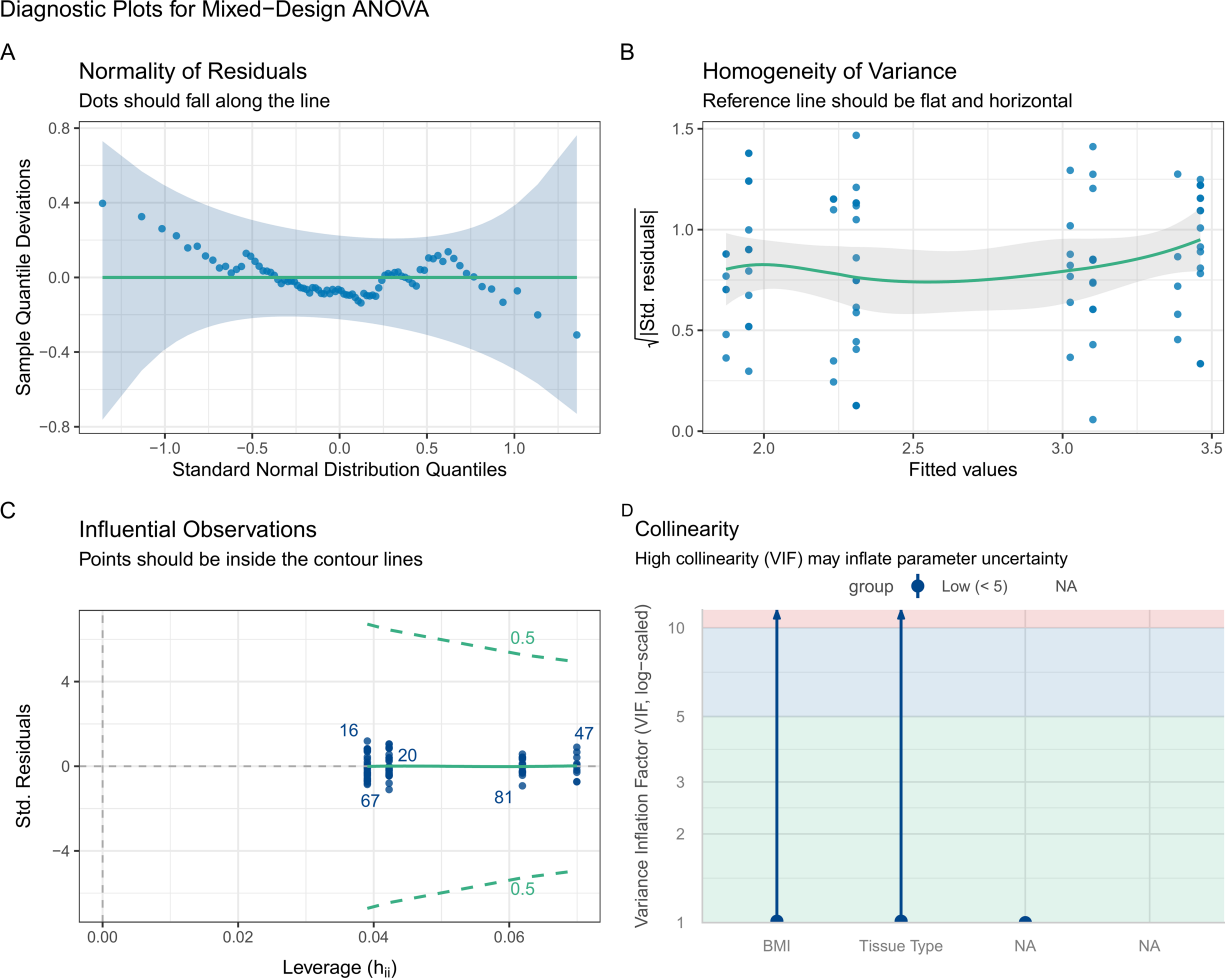
Diagnostic Plots for Mixed-Design ANOVA. The diagnostic plots assess the assumptions of the mixed-design ANOVA model, including **(A)** normality of residuals, **(B)** homogeneity of variance, **(C)** influential observations, and **(D)** collinearity among predictors.

Assumptions for the mixed-effects model were met. Diagnostic checks confirmed normality and homoscedasticity of residuals (**Figure 6**), the absence of influential outliers, and no significant multicollinearity (all VIF< 5).

## Discussion

### Summary of main findings

The primary contribution of this study is the robust, quantifiable evidence demonstrating that PIs are characterized by a profound increase in local skin thickness. This effect was remarkably strong, with the presence of a PI lesion accounting for approximately half the total variance in skin thickness ( $\eta_{g}^{2}=0.500$, *p* < 0.001) relative to adjacent healthy tissue. This finding solidifies localized skin thickening and structural alteration as a definitive hallmark of pressure-induced pathology. Extending beyond this main effect, our analysis revealed that a patient’s intrinsic risk status acts as a critical modulator. Specifically, the pathological thickening was significantly more pronounced in high-risk individuals, suggesting that higher baseline vulnerability may predispose patients to a more severe tissue response. In contrast, common demographic factors such as BMI, age, and gender did not show significant influence, indicating that their roles are likely secondary to the immediate pathological process and the patient’s pre-existing risk profile.

### Interpretation and mechanistic discussion of results

The observed skin thickening in PI sites is biologically plausible. Pathologically, PIs primarily develop due to ischemia–reperfusion injury, microvascular compromise, and deep tissue deformation, which often occur before superficial edema can be measured. Skin thickening may arise from two distinct but overlapping processes: acute inflammatory swelling in the early injury stage and subsequent chronic fibrotic remodeling during dysregulated tissue repair. These two processes represent different stages of tissue injury and healing, and both can contribute to the increased skin thickness detected by high-frequency ultrasound (Wang et al., 2024; Emaminia et al., 2025). Therefore, the thickness measured by ultrasound serves as an objective, quantifiable biomarker of the tissue’s ongoing pathological response to mechanical stress (Marcus et al., 2021).

Critically, this response is not uniform; it is significantly modulated by the patient’s systemic vulnerability. We hypothesize that the pro-inflammatory, microvascularly compromised state (Sebastian et al., 2023; Paramasivam et al., 2024) in high-risk individuals amplifies the local response to ischemic insults, leading to the exaggerated thickening we observed. This interpretation is supported by the marginally significant interaction effect (*p* = 0.082), which, given its moderate effect size ( $\eta_{g}^{2}=0.050$) and our study’s limited power, likely represents a clinically important signal rather than a null finding (Ahmed and Butt, 2025; Fu et al., 2025). This suggests the magnitude of tissue alteration is contingent on the patient’s overall frailty, a hypothesis warranting confirmation in larger studies (Kang, 2021).

### Comparison with existing literature

Our finding of significant skin thickening in PI lesions aligns with previous ultrasound studies like Wang et al. (2025) (Hanlon et al., 2023) but advances the field in a critical way. By employing a within-subject design comparing lesions to adjacent healthy tissue, we unequivocally attribute this thickening to the pathology itself, controlling for inter-patient variability (Wang et al., 2025). Furthermore, while diagnostic studies like Ren et al. (2025) (Bykov et al., 2020) have demonstrated the predictive power of ultrasound, our work provides the mechanistic rationale: the profound structural change we quantified ( $\eta_{g}^{2}=0.500$) is the biological basis for ultrasound’s diagnostic efficacy. We also address apparent contradictions in the literature, including reports of tissue thinning versus thickening in PI studies. Our interpretation that tissue thinning may represent pre−ulcerative atrophy in high−risk skin prior to visible injury, while thickening reflects established lesions, remains plausible (Kwek et al., 2023; Ren et al., 2025). However, we acknowledge that the literature on ultrasound evaluation of PI is still evolving, and inconsistencies across studies may arise from multiple key methodological factors, including differences in ultrasound frequency and resolution, variability in measurement protocols, differences in anatomical measurement sites, and variation in the timing of ultrasound assessment relative to injury development. A more comprehensive consideration of these factors provides a balanced explanation for divergent findings in the literature.

Methodologically, our use of a mixed-design ANOVA (Yang et al., 2021; Steinhilber et al., 2024) allowed a more nuanced analysis than is typical, partitioning variance to reveal that a patient’s systemic risk status moderates the local tissue response. This novel finding—that high-risk status appears to potentiate PI-induced thickening—moves the field beyond simple detection towards a more personalized understanding of PI pathophysiology, echoing calls from reviews like Tzen et al. (2025) (Pfister, 2024). Although the interaction effect was only marginally significant, likely due to limited statistical power (Nakagawa et al., 2024; Tzen et al., 2025), it provides a strong, hypothesis-generating signal. This underscores the need for future, larger-scale studies, perhaps integrating complementary techniques like shear wave elastography (Mansur et al., 2021) (Carey et al., 2023), to confirm how systemic health dictates the severity of local tissue injury (Howlett et al., 2021; Mansur et al., 2021).

### Study limitations

This study’s primary limitation is its modest sample size (N = 42), which resulted in low observed power (0.56). This renders our findings preliminary and any null results inconclusive due to a potential for Type II errors (Davinelli et al., 2021). Furthermore, the cross-sectional, single-center design prevents causal inference and limits the generalizability of our results (Carlin et al., 2024). Finally, the perilesional tissue used as the “healthy” control may still exhibit subclinical pathological alterations, given that pressure injury–related tissue damage may extend beyond the visually identifiable lesion boundary.

Therefore, future research should prioritize adequately powered, longitudinal, multi-center studies employing standardized, multi-rater protocols. Such a design is essential to establish causality, validate skin thickening as a predictive biomarker, and ensure the robustness of our findings. Technologically, integrating our thickness measurements with advanced methods like shear wave elastography (SWE) could yield a more comprehensive tool for PI risk stratification and prognosis (Aslanyan et al., 2023).

### Clinical and research implications

This study demonstrates that skin thickness, measured by HFUS, is a robust and objective biomarker of the structural changes in PI tissue (Liu et al., 2024). Using a rigorous within-subject design, we definitively show that PI tissue is significantly thicker than adjacent healthy skin, a difference primarily driven by the pathological tissue state itself. Critically, we identified that a patient’s systemic risk status is a key modulator of this response, with high-risk individuals exhibiting a trend towards more pronounced thickening. In contrast, demographic factors like BMI, age, and gender had a negligible influence (Tan et al., 2025).

The clinical implications are significant. HFUS provides an objective method to augment subjective visual assessment (Tusar et al., 2025), quantify deep tissue involvement (Wei et al., 2025), and potentially monitor treatment efficacy. The finding that high-risk patients may mount an exaggerated tissue response suggests a paradigm shift in their management (National Pressure Injury Advisory Panel et al., 2025). For these vulnerable individuals, HFUS could be strategically deployed for early detection of pathological thickening, enabling preemptive interventions before irreversible skin breakdown occurs. Future work must now focus on validating these findings in longitudinal, multi-center trials and integrating HFUS with other modalities like elastography to create a multifaceted tool for PI risk stratification and management (Alderden et al., 2025).

### Future research directions

We recommend integrating HFUS into high-risk patient assessments as an objective adjunct to clinical evaluation (Albano et al., 2023). Effective implementation requires the urgent development of standardized protocols and interprofessional training (Tang et al., 2023). Critically, for widespread adoption, professional societies must advocate for new reimbursement codes, as financial viability is the primary barrier. A strategic roadmap, beginning with pilot studies and progressing to multicenter validation, is necessary to translate this evidence into a transformative clinical tool that improves PI prevention and management (Thawani et al., 2023).

## Conclusions

In conclusion, HFUS quantitatively demonstrates significant structural differences in skin thickness between established PI lesions and adjacent healthy tissue, confirming skin thickening as a reliable marker of established injury. For future clinical applications, we propose that high-frequency ultrasound may be used for predictive monitoring to detect early subclinical tissue changes before visible PI develops, enabling timely preventive interventions.

## Data Availability

The raw data supporting the conclusions of this article will be made available by the authors, without undue reservation.

## Supplementary material

Supplementary Figure 1 Mixed Correlation Matrix of Key Variables. The mixed correlation matrix displays the correlation coefficients among Age, BMI (Body Mass Index), Pressure Injury Risk Status, Sex, Skin Thickness, and Tissue Type. Significant correlations are indicated with asterisks: ***p< 0.001; **p< 0.01; *p< 0.05.
