# Supplementary material for: Skin thickness alterations in pressure injury tissue: insights from high-frequency ultrasound and mixed-design analysis of variance
Source: Front Physiol. 2026 Jun 3;17:1824326. doi: 10.3389/fphys.2026.1824326 (PMC13272420; doi:10.3389/fphys.2026.1824326)
Supplement: Supplementary Figure 1 — Mixed Correlation Matrix of Key Variables. The mixed correlation matrix displays the correlation coefficients among Age, BMI (Body Mass Index), Pressure Injury Risk Status, Sex, Skin Thickness, and Tissue Type. Significant correlations are indicated with asterisks: ***p< 0.001; **p< 0.01; *p< 0.05. [file SupplementaryFile1.docx]

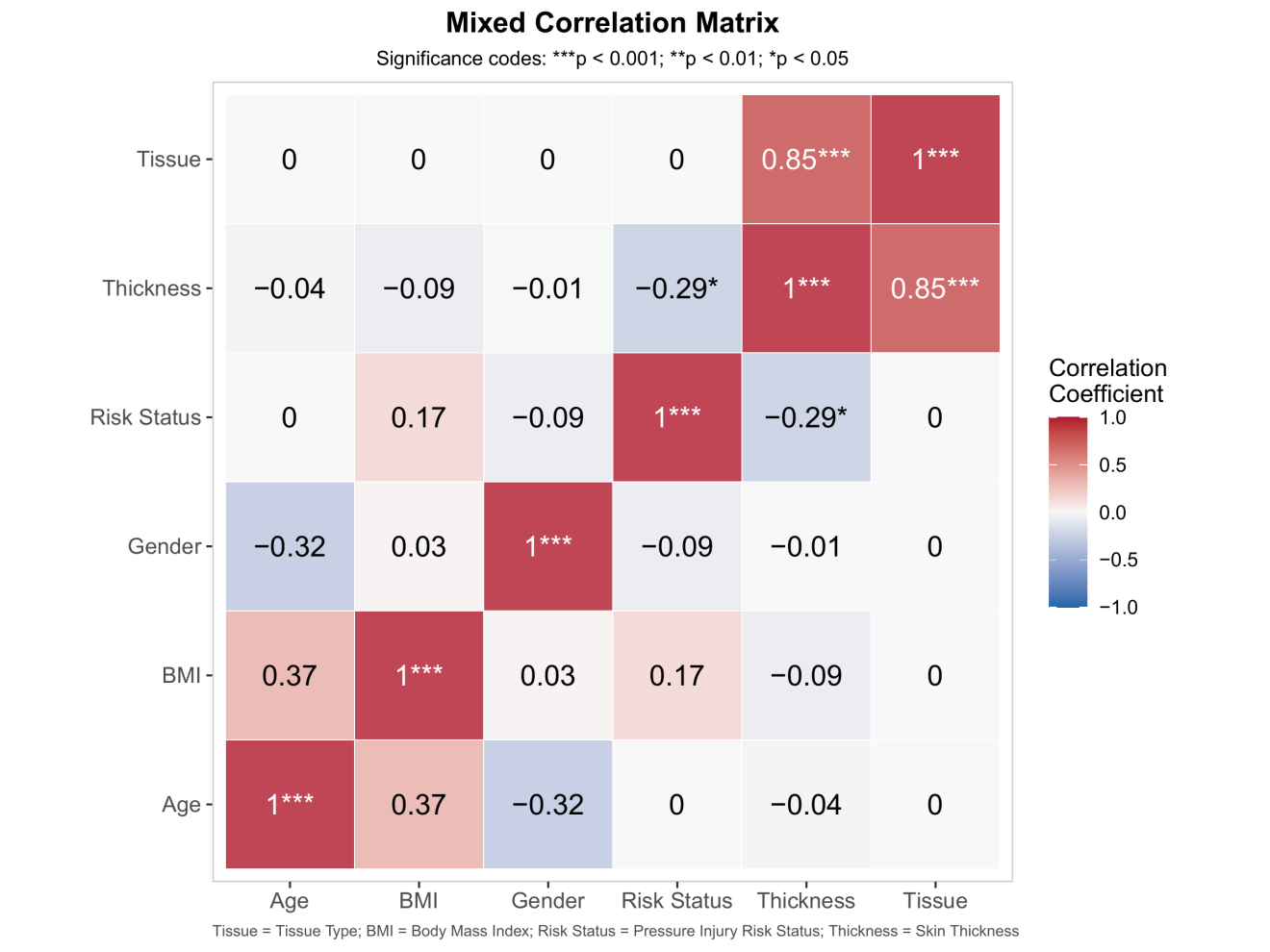


Supplementary Figure S1: Mixed Correlation Matrix of Key Variables.The mixed correlation matrix displays the correlation coefficients among Age, BMI (Body Mass Index), Pressure Injury Risk Status, Sex, Skin Thickness, and Tissue Type. Significant correlations are indicated with asterisks: ***p < 0.001; **p < 0.01; *p < 0.05.
